# Supplementary material for: A redefined InDel taxonomy provides insights into mutational signatures
Source: Nat Genet. 2025 Apr 10;57(5):1132–41. doi: 10.1038/s41588-025-02152-y (PMC12081297; doi:10.1038/s41588-025-02152-y)
Supplement: Supplementary file 1 — Supplementary Fig. 1 and Supplementary Note. [file 41588_2025_2152_MOESM1_ESM.pdf]

nature genetics

Supplementary information https://doi.org/10.1038/s41588-025-02152-y

A redefined InDel taxonomy provides insights into mutational signatures

In the format provided by the authors and unedited

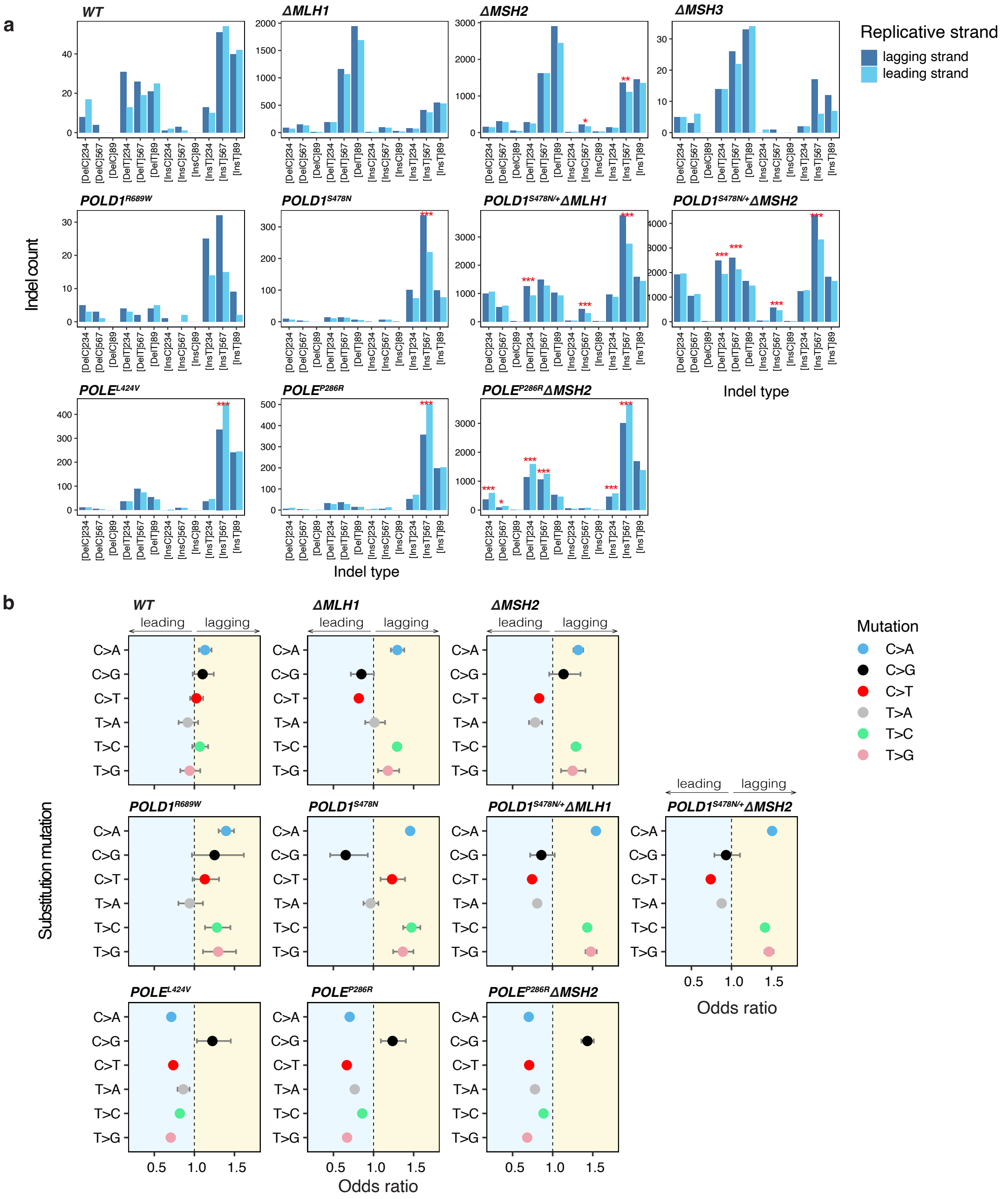

Supplementary Fig. 1: Replicative strand asymmetry analysis. **a**, Replicative strand bias analysis of aggregated indels per gene-edits. **b**, Replicative strand bias analysis of aggregated substitutions per gene-edits. Binomial test adjusted *P* values for significance: *$P<0.05$; **$P<0.01$; ***$P<0.001$. See Supplementary Table 6 for detailed results and exact *P* values.

Supplementary Note 1: Indel Segmentation

Mechanisms of indel formation often involve insertion or deletion of nucleotide sequence located 3’ to the event[1]. Previous approaches to indel classification have used this property to stratify indels into mechanistically associated groups by determining the repetitiveness of the inserted / deleted sequence with respect to the 3’ sequence context[2]. However, we and others have observed that significant ambiguity exists in binary threshold-based classification schemes (*i.e.*, “repeat-mediated” vs “microhomology-mediated”). Specifically, in this work we find that restricting indel classification to predefined and mechanistically associated categories can obscure and collapse mutational signatures produced through engineered genetic perturbations (See main manuscript Fig. 1, Extended Data Fig. 1).

Previous approaches quantifying indel repetitiveness have used different measures of repetition dependent on the mechanistically associated class[2]. For example, in early mutational signature analyses of indel mutagenesis, microhomology was determined as the shared prefix between an indel and the 3’ sequence context, while repeat length was separately determined as the number of times the indel could be found in the 3’ sequence context. This is predicated on the convention that the indel variants be left-aligned with respect to the reference genome, and the variant sequence is represented parsimoniously, hence the repeat and/or microhomology flanking an indel is found downstream in the 3’sequence.

In this work, we sought a unified and reproducible strategy for determining the relationship between an indel and the 3’ sequence context. Here, we outline this strategy, which we term “indel segmentation”, which explicitly identifies the smallest prefix sequence motif that displays a maximal repetitive relationship with the 3' sequence context and within the indel itself. This provides a standardized approach for relating indel sequence to the 3’ sequence context. Using these segmentations, we then group indels into a set of new, non-overlapping sub-categories, or “channels”, for mutational signature analysis. Below, we describe the indel segmentation process, as well as the resulting procedure for constructing the final 89-channel format used in this manuscript from the set of observed segmentations across pan-cancer cohorts.

Preprocessing indel variant calls

We follow the standard convention of normalizing indels through left-alignment (VCF standard, https://samtools.github.io/hts-specs/VCFv4.2.pdf). Because our method depends on comparing indel sequence, we suggest indels be called with high confidence (VAF > 0.2). Within our described framework of small indels, we focus specifically on insertions, deletions, and complex indel events with lengths < 100bp; homopolymer repeat and polynucleotide repeat with units < 10 because of the high error rates in mutation-calling algorithms at long, simple repeats; and a 3’ sequence context of up to length 200bp. Insertions with microhomology are segmented and classified using our unified framework into various non-overlapping “insertion” and “complex” channels for we currently do not have a designated category for these complex events. This is

because templated insertions are rare, and often complex (*i.e.*, they are accompanied by deletion at the junction). Majority of them would have been overlooked or mis-annotated using standard indel variant callers[3]. Furthermore, they could also be templated from inverted repeats (both left and right flanks) and not be immediately at the indel junction, and unaccompanied by deletions. Due to the complexity of these events, they are best characterized by specialized algorithms[3,4]. We note that our segmentation approach generalizes to indels of all lengths, including 1bp indels where the prefix is unambiguous.

Algorithm for segmenting indels

The following procedure for indel segmentation is applied to each indel variant:

1) Starting from the 1st position of an indel, generate multiple 'units', each representing different prefixes of length *k* (*k* from 1 to the length of the indel) bases from the first base of the indel sequence.

   E.g., for the indel | 3' sequence pair:

   `ATCATCGA | ATCATCATCGGGGG`

   The possible units are:
   - `A`
   - `AT`
   - `ATC`
   - `ATCA`
   - `ATCAT`
   - `ATCATC`
   - `ATCATCG`
   - `ATCATCGA`

2) For each "unit", determine how many exact tandem repeats of this unit exist in the 3' sequence context.

3) For each "unit", determine the number of exact tandem repeats of the unit within the indel, not including the first occurrence, starting from the end of the prefix sequence.

4) Term the remaining length of the indel the "spacer", which separates the repetitive region within the indel and the repetitive region in the 3' sequence context.

Each segmentation, therefore, has four associated values:

1) Unit length (**U**): The length of the "unit" in base pairs (bp), and associated nucleotide sequence.

2) Internal Repetition (**IR**): The number of times the unit sequence is tandemly repeated within the indel, and the nucleotide sequence of those repetitions.

3) Spacer (**S**): The length in bp of the remaining sequence between the last tandem repetition of the unit within the indel, and the end of the indel, as well as the associated nucleotide sequence.
4) Three Prime Repetition (**TR**): The number of times the unit sequence is tandemly repeated in the 3' sequence context, and the associated nucleotide sequence.

For each indel of length *k*, there are *k* possible segmentations corresponding to the *k* different prefixes beginning at the first position of the indel, each represented by the above four values (U, IR, S, TR). For example, in the above 8 bp indel and 3' context pair, we have 8 possible units and therefore, 8 possible segmentations. Different segmentations capture different relationships between an indel and its associated 3' context. To capture the relationship most relevant for indel classification, we use two criteria based on previous work stratifying indels into mechanistically associated categories[2,5,6].

Criterion 1: We assume that repetitive motifs drive and enhance indel mutagenesis[1].

Criterion 2: In the absence of additional information, we assume the minimally sufficient representation of the unit is the most informative.

We then select the segmentation that is most concordant with the above criteria as the optimal segmentation for the indel. Practically, this is equivalent to trying to optimize for the following properties, in the following order:

1) Maximize 3' repetition, **TR** → We prioritize a segmentation where the unit is highly repeated in the 3' region, because this repetition is the most likely mechanistically relevant, following *Criterion 1*.

2) Maximize internal repetition, **IR** → As for 3' repetition, maximizing internal repetition is consistent with *Criterion 1*.

3) Minimize spacer length, **S** → Minimizing the number of bases between the repetitive region of the indel and the 3' region in turn maximizes the length of the repetitive region, consistent with *Criterion 1*.

4) Minimize unit length, **U** → The smallest repetitive unit that yields a segmentation that meets the above criteria is also the minimally sufficient unit, in accordance with *Criterion 2*.

We sort all segmentations per indel using the above preferences in the order they are listed and select the top segmentation as the most optimal with respect to the two criteria, and therefore the most representative and biologically meaningful of the process of indel formation. To concisely describe the optimal segmentation – it is the smallest possible unit that has the highest 3' repetition, internal repetition, and smallest spacer, out of all possible segmentations.

Considering the earlier example:

`ATCATCGA | ATCATCATCGGGGG`

The selected segmentation is:

Unit / Repetitive motif (U)	Internal Rep (IR	Spacer (S)	3' Repetition (TR)
ATC	ATC	GA	ATCATCATC
3bp	1 Rep	2bp	3 Reps

It is important to note that when one of the four key values are zero, the sorting procedure then prioritizes maximizing or minimizing the subsequent value, following the above priority indicated in the sorting criteria. This allows indels that may have no 3' repetition to still be assigned a unique and an optimal segmentation with a minimally sufficient unit and maximal internal repetition. In cases where there is no repetition at all, the priority of minimizing the length of the spacer results in maximizing the unit length by counting the entire indel as the unit.

Accounting for original sequence context (Original Repetitions)

For an insertion or a deletion of perfectly repetitive sequence with no spacer (S = 0), the original number of times the unit is repeated within the DNA sequence before mutagenesis reflects the sequence context for mutagenesis and is, therefore, biologically relevant.

For deletions, this number of repetitions is equal to the number of 3' repetitions (TR), plus the number of times the unit is repeated within the indel (internal repetition, IR) plus the first occurrence of the unit (1). Thus, for deletions, we calculate an adjusted value which we denote as "Original Repetitions" or "**R**".

(Deletions) `R = TR + IR + 1`

For insertions, we do not need to adjust the number of repetitions, as the number of 3' repetitions (TR) accurately represents the number of times the unit was present in the DNA prior to mutagenesis. Therefore, for insertions:

(Insertions) `R = TR`

Finally, for all indels where repeats are not perfect, (*i.e.*, S > 0), or complex indels, the number of 3' repetitions remains biologically relevant. Hence, for all other mutations:

(All others) `R = TR`

The following pseudocode summarizes the calculation of **R**:

```
if S = 0:
        if deletion:
              R = TR + IR + 1
        else if insertion:
              R = TR
        else:
              R = TR

if S > 0:
        if insertion:
                R = TR
        if deletion:
                R = TR
        else:
                R = TR
```

Accounting for microhomology length (Mh-length)
For indels where the associated segmentation indicates a non-zero length spacer (*i.e.,* S > 0), we additionally calculate the length of the unit and internal repetition as the length of microhomology or "**Mh**" between the indel and the 3' sequence context as:

$$Mh = \min(U + IR \times U , TR \times U)$$

Where we take the minimum length of the two regions as the minimally sufficient, and exact matching, region of microhomology.

Reference implementation
A reference implementation of the above algorithm is provided at https://github.com/Nik-Zainal-Group/indelsig.tools.lib, as well as reproduced here (See, *Reference implementation*).

Constructing indel channels using indel segmentations

With the optimal indel segmentations derived following the procedure outlined above, the associated values (**R**, **Mh**, **U**, *etc.*) as well as the surrounding sequence context can be used to construct "channels", or sub-groups of indel variants sharing common properties. While previous approaches to indel sub-grouping and channel construction have used combinatorial enumeration of indel-associated values (*e.g.,* indel motif length from 1 – 5 bp or more, number of repeats from 0 – 6 bp or more), the resulting mutational signatures are often imbalanced (Fig. 1, Extended Data Fig. 3). We were motivated to take a different approach, having observed in experimental data that an imbalance leads to inaccurate detection of PRRd-associated signatures in isogenic gene edits (Fig. 2).

We instead sought to construct a set of channels that distributed signal more evenly across channels, while maintaining power for signature analyses. To do so, we first segmented indels from three independent pan-cancer cohorts consisting of 18,522 samples from ICGC/TCGA[7], the Hartwig Medical Foundation consortia[8] and Genomics England 100kGP[9], spanning 29 cancer tissue types and representing a significantly majority of high-quality cancer whole genome data presently available. We then enumerated all discovered segmentations, incorporating 1 bp of 5' and 3' sequence context for 1 bp indels. This yielded a set of 476 initial channels that captured the diversity of mutational outcomes similarly across datasets (Extended Data Fig. 3e).

Given the average number of indels per sample (~ 100 – 1000 indels, varying by tissue type/cohort), we sought to further merge these 476 channels to produce a reduced representation that remained informative for signature analysis, while increasing *signal-to-noise* per sample. We achieved this by defining multiple partitions of segmentation values (**R**, **Mh**, **U**, *etc.*) and merging channels within these partitions into new, aggregated channels. Subsets of multiple initial channels were aggregated to single channels, producing a final 89-channel set where an indel could be unambiguously and uniquely assigned to a single channel.

A detailed description of each channel, including the specific boundaries on segmentation values and examples of candidate indels, is presented in Supplementary Table 4. A schematic of this merging process is depicted in Fig. 2a and Extended Data Fig. 3e,f. We additionally describe below the curation process for establishing which channels could be merged. Our overarching objective in merging was to increase per-sample *signal-to-noise*, while balancing signal across channels.

Merging subsets of initial 476 channels to produce an 89-channel set
Broadly, we observed indels fell into 5 classes: 1 bp insertions and deletions, ≥ 2 bp insertions, ≥ 2 bp deletions of exact repeats (**S** = 0), ≥ 2 bp deletions with a spacer (**S** ≥ 1), and complex indels. Within these classes, we identified relevant subgroups for which data indicated partitions could be established and channels aggregated. These are as follows:

1) 1 bp insertions and deletions

Across all datasets, with or without hypermutators, 1 bp insertions and deletions account for, on average, ~30 – 55% of all available signal per sample (Extended Data Fig. 3c,d). In our initial 476 channel set, 1 bp indels were translated to their associated pyrimidine context (*i.e.,* C or T) and their 5' and 3' sequence context, as well as the number of original repeats (**R**, equivalent to homopolymer tract length, since the unit for 1 bp indels is a single base inserted or deleted**)**, used as criteria for channel aggregation.

With respect to 5' and 3' sequence context, we considered if retaining this information elided specific outcomes (for example, T deletions at homopolymer tracts adjacent to Cs) versus the increase in signal from ignoring it. With respect to homopolymer tract length / **R**, we examined the distribution of **R** for insertions and deletions and attempted to select boundaries that matched the observed densities. We applied this procedure separately for T and C insertions and deletions as follows:

a) T insertions / deletions:
   Across datasets, T insertions and deletions displayed specific and varied patterns when stratified by 5' and 3' sequence context. With the abundance of signal, we considered all 5' and 3' single nucleotide combinations around a central homopolymer variant.

   Examining the frequency of homopolymer tract lengths / **R** for T insertions and deletions, we found the density roughly separated into three regions: 0 – 4, 5 – 7, and 8 – 9. We therefore additionally grouped T indels into 3 homopolymer tract length / **R** groups: (**R**: 0 / 1 ≤ **R** ≤ 4, 5 ≤ **R** ≤ 7, 8 ≤ **R** ≤ 9).

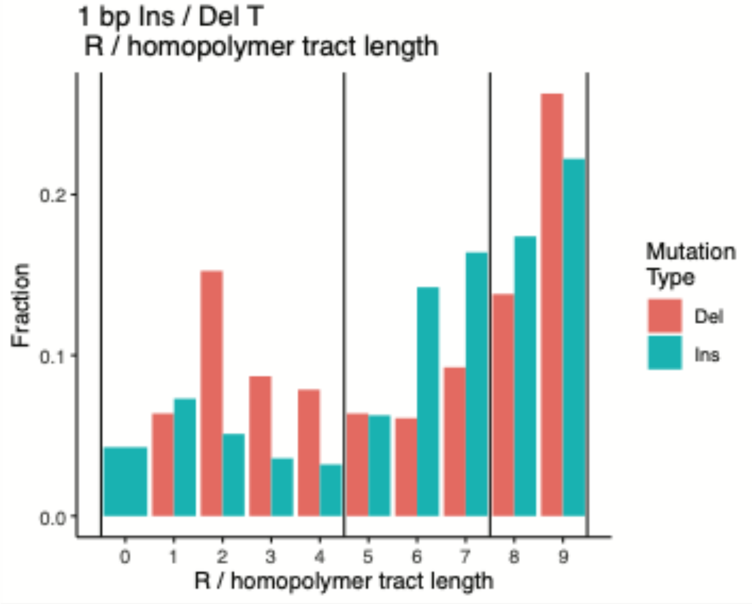

Together, this produced (3 **R** groups) × (9 of 5' and 3' context pairs) = 27 channels for T insertions and T deletions each.

b) C insertions:
We observed C insertions were relatively infrequent across datasets (Extended Data Fig. 3e) and thus sought to concentrate signal into a smaller set of channels than for T insertions / deletions. We therefore only considered sequence context for non-templated insertions (**R** = 0) at specific sequence contexts (5'A / 3'A, and 5'A / 3'T), producing two channels.

We then stratified the remaining C insertions by number of original repeats (**R**: $0 \leq$ **R** ≤ 3, $4 \leq$ **R** ≤ 6, and $7 \leq$ **R** ≤ 9), producing 3 channels.

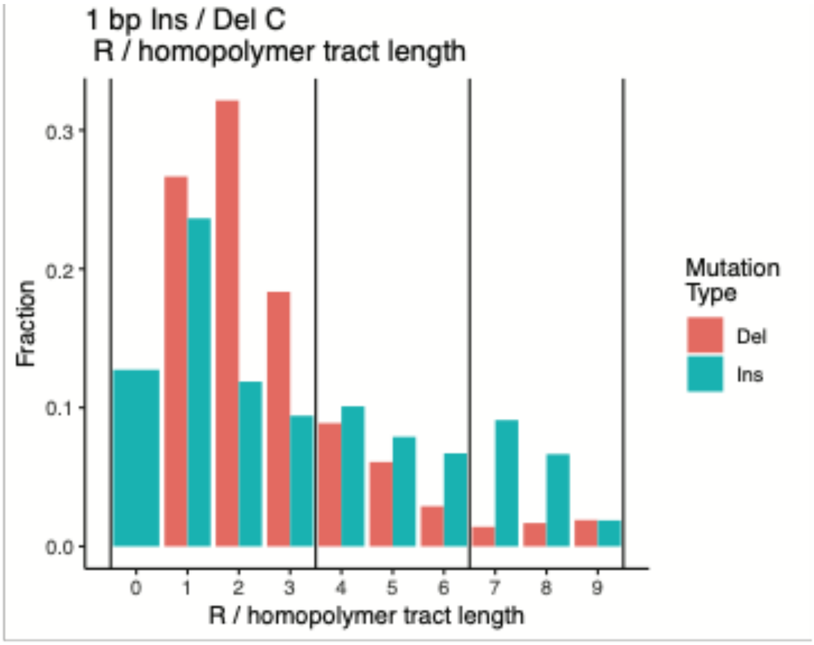

In total, this resulted in 5 channels for C insertions.

c) C deletions:
In the datasets examined, C deletions were more frequent than C insertions (Extended Data Fig. 3e) but were still less frequent than T indels and necessitated channel aggregation to increase signal. The increased frequency, however, allowed for more granular dissection of mutational outcomes by reintroducing sequence context. We noted that mechanistically, the 3' sequence context is particularly relevant for deletions, with 3'A and 3'T contexts occurring most frequently.

Given the observed density in the plot above, we therefore constructed channels describing C deletions at 1, 2, 3, or 4 – 5 repeats adjacent to A or T nucleotides in the 3’ context, producing a total of 4 × 2 = 8 channels.

For the remaining C deletions at shorter homopolymers (1 ≤ **R** ≤ 5) at 3’C / 3’G sequence contexts, we aggregated signal into a single channel. Finally, we aggregated C deletions at longer homopolymers (**R** > 5) into a single channel, irrespective of the sequence context to maximize signal. In total, this produced 8 + 2 = 10 channels for C deletions.

2) ≥ 2 bp insertions:
For ≥ 2 bp insertions, which are relatively infrequent across the datasets considered in this study, (~ 8 – 10% of signal, varying by dataset), we could stratify events by any segmentation values (**R**, **Mh**, **U**, *etc.*). However, rather than enumerate all possible combinations, we instead delineated channels based on the repetitiveness of the original sequence context. This resulted in channels for R = 0 (*non-templated* insertions), **R** = 1 (*tandem duplication* events), 2 ≤ **R** ≤ 4 (*moderately repetitive* regions) and **R** ≥ 5 (*highly repetitive* regions).

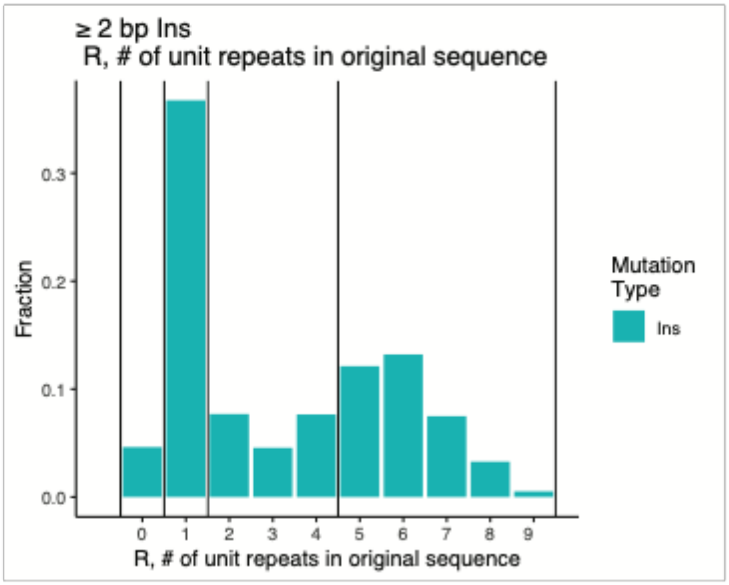

Where additional stratification was possible without compromising *signal-to-noise*, we partitioned indels by length, with 2 – 4 bp (short) and ≥ 5 bp (long) as separate channels. The decision to do so was, again, motivated by increasing resolution where possible without sacrificing power. This was only possible for **R** = 0 and **R** = 1 classes, resulting in a total of 2 (**R** = 0, **R** = 1) × 2 (short, long) + 2 (2 ≤ **R** ≤ 4, **R** ≥ 5) = 6 total channels for ≥ 2 bp insertions.

3) ≥ 2 bp deletions of exact repeats (**S** = 0)

For ≥ 2 bp deletions without a spacer (**S** = 0), and therefore consisting of exact repeats, we similarly stratified variants by repetitive content. For deletions where only one copy of the relevant unit existed in the original sequence (**R** = 1, or non-repetitive), we found that indels partitioned roughly based on length.

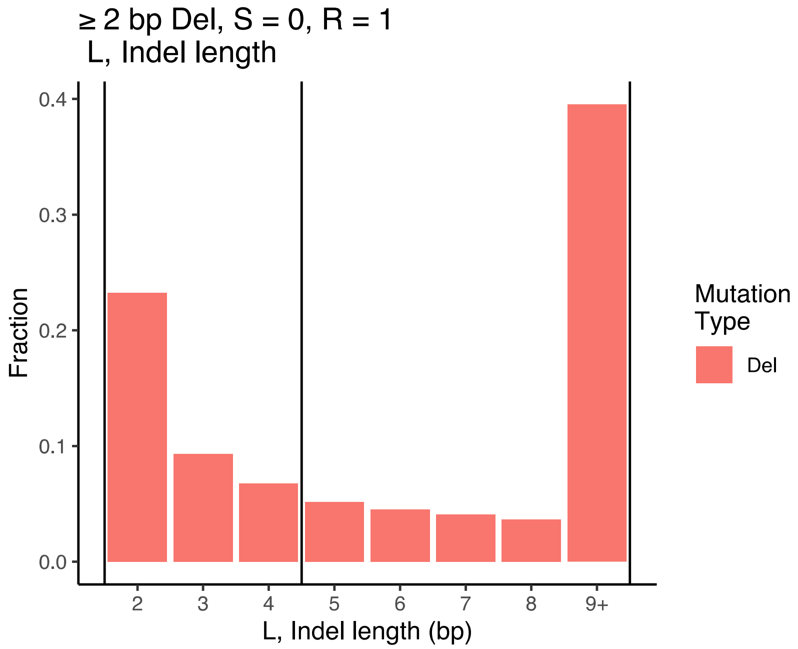

We therefore stratified **R** = 1 events by indel length, 2 – 4 bp (short) and ≥ 5 bp (long), producing two channels.

For deletions with multiple copies of the relevant unit in the original sequence (**R** ≥ 2), the unit is the minimal repetitive unit, likely corresponding to complex "n-mer" (*e.g.*, 3-mer (`ATGATGATG`…), 4-mer (`ATGCATGC`…)) repetitive sequence. We therefore examined the unit to determine effective boundaries:

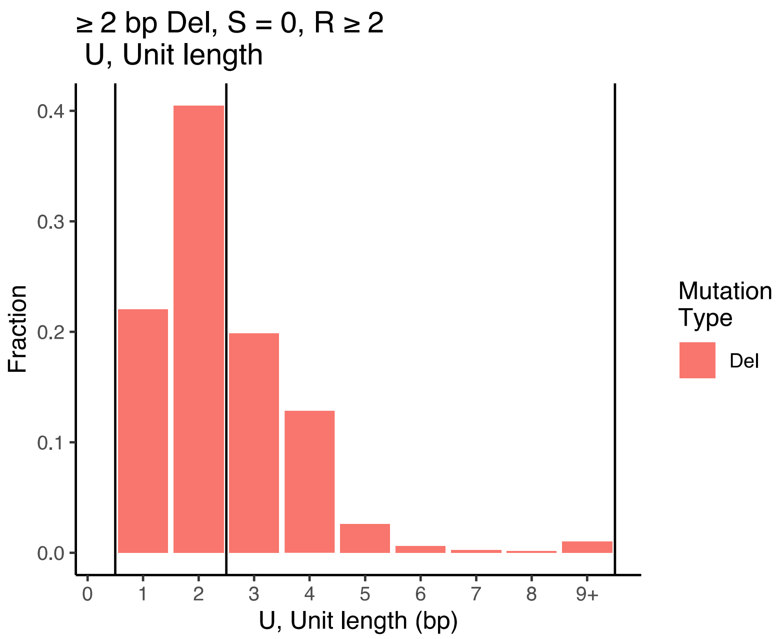

From the observed density, we determined units could be partitioned into two groups, short (1 ≤ **U** ≤ 2 bp), or long (**U** ≥ 3 bp).

For short unit deletions, we then found we could partition indels further by degree of repetitiveness (2 ≤ **R** ≤ 4, 5 ≤ **R** ≤ 9), while for long unit deletions, partitions **R** = 2 and 3 ≤ **R** ≤ 9 better captured and balanced the observed density:

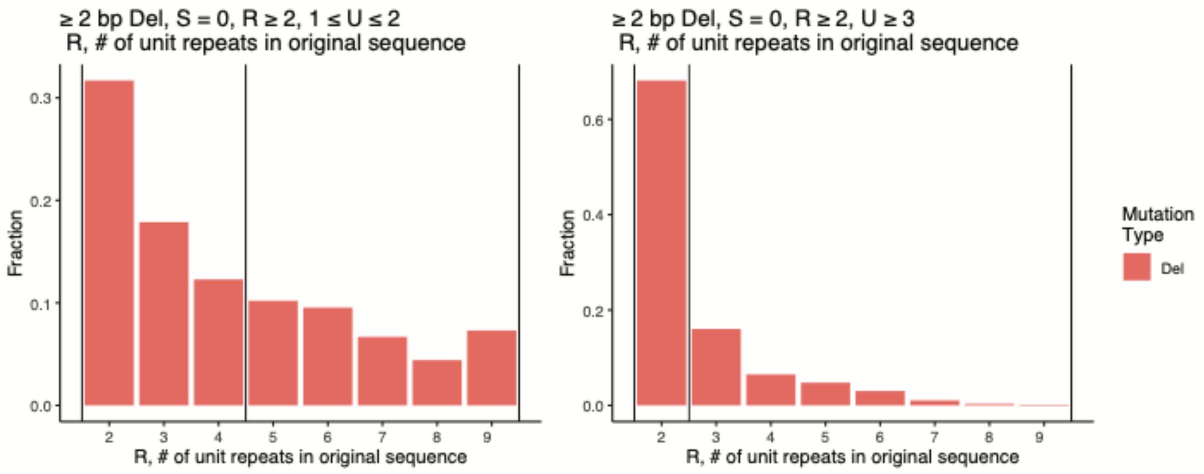

In total, this produced 2 non-repetitive (**R** = 1, short and long) + 2 (**U:** short, 1 ≤ **U** ≤ 2, **R:** 2 ≤ **R** ≤ 4, 5 ≤ **R** ≤ 9) + 2 (**U:** long, **U** ≥ 3, **R:** 2 ≤ **R** ≤ 4, 5 ≤ **R** ≤ 9) = 6 channels for ≥ 2 bp deletions with **S** = 0.

4) ≥ 2 bp deletions with a spacer (**S** ≥ 1):

For ≥ 2 bp deletions where a spacer was identified during segmentation, we reasoned that the lack of contiguous exact repeats between the indel and the 3' context reduced the biological value of considering **R**, and instead motivated consideration of length of putative microhomology (**Mh**).

Examining the distribution of indel length for indels with **S** ≥ 1, we observed most could be broadly clustered as short (2 – 5 bp) and long (≥ 6 bp). The increased density observed for long indels is derived from the extremely long tail of indel lengths, ranging from 6 bp up to 100 bp.

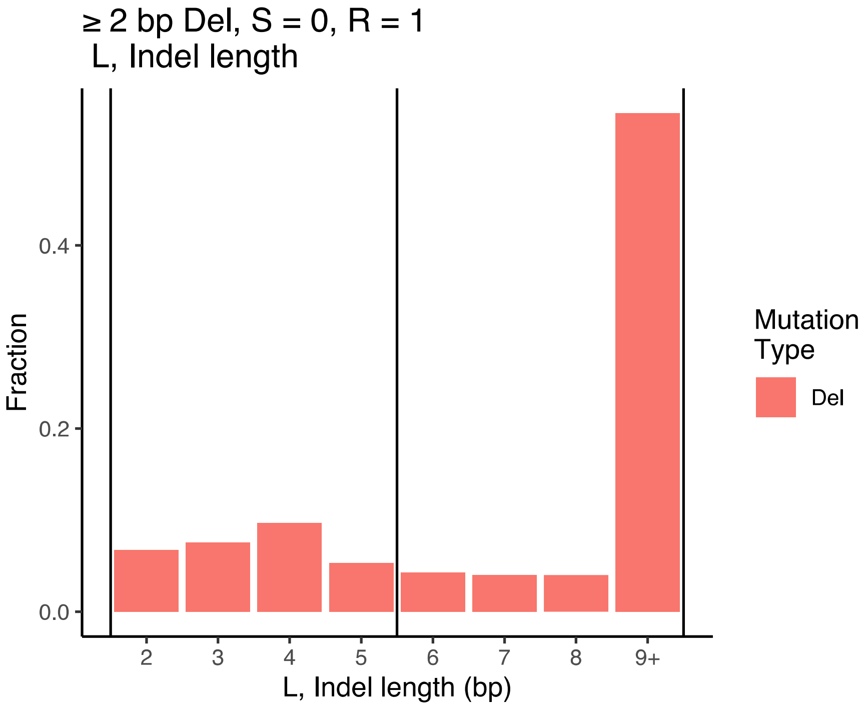

Examining the distribution of **Mh** stratified by indel length (short vs. long), we observed shorter **Mh** lengths were more frequent (**Mh** ≤ 4), and could be partitioned around single values (*i.e.*, **Mh** = 1, **Mh** = 2, *etc.*) due to the increased signal.

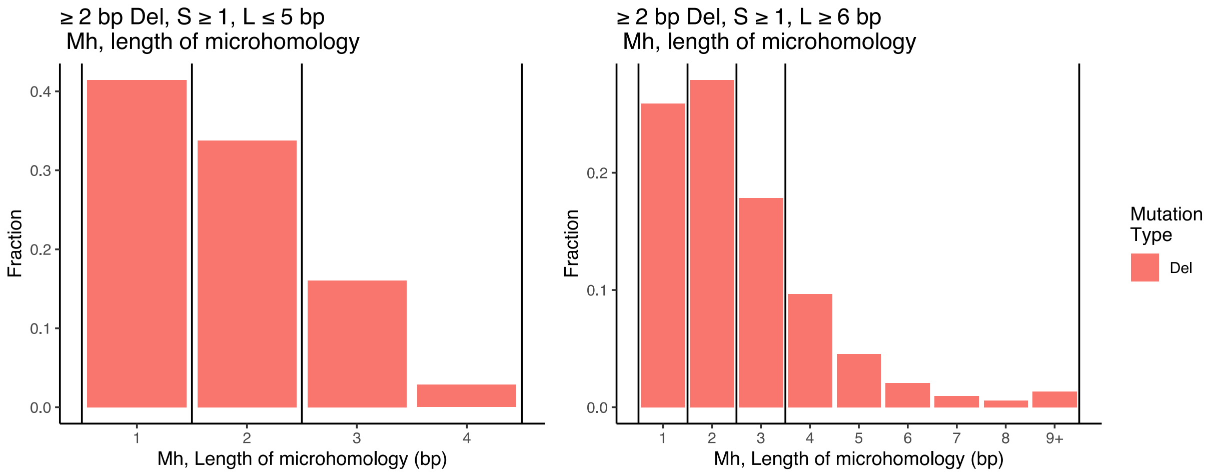

We therefore decided to maximize resolution by constructing channels based on the minimal to maximal amount of microhomology given a specific indel length. For example, an indel of length 5, the maximum of the "short" indels we observed, would have at most 4 bp of microhomology, or **Mh** = 4 bp (if **Mh** = 5 bp, it would be an exact repeat).

With this approach, we identified 3 partitions for short indels (**Mh** = 1 bp, **Mh** = 2 bp, and **Mh** ≥ 3) and 4 partitions for longer indels (**Mh** = 1 bp, **Mh** = 2 bp, **Mh** = 3 bp, **Mh** ≥ 4 bp).

Together, this resulted in 7 channels for ≥ 2 bp deletions, with a spacer (**S** ≥ 1).

5) Complex indels

Though complex indels can be segmented, their frequency is low. Hence, we did not enumerate observed segmentation values in our initial set of 476 channels. Therefore, all complex indels were grouped in a single channel.

In total, the set of 89 channels constructed following the above procedure, and using the exact partitions as described, could effectively represent the spectrum of indel variation observed in this study (Extended Data Fig. 3b-f). Further, this set more evenly distributed signal across all channels as compared to COSMIC-83 (Extended Data Fig. 3c, d). This highlights the relevance of building channels that adequately capture the observed variants in a dataset.

Additionally, while we note that alternative partitioning procedures are possible and remain a topic for further study, the current 89-channel set considered in this study was designed for, and performs adequately across, a range of tumor samples from diverse tissues and patients that comprise a large majority of available tumor genomes presently available to the boarder research

community. Thus, we contend that our newly proposed 89-channel indel taxonomy should be generalizable across pan-cancer datasets and finds wide-spread adoption for investigating indel mutagenesis in the framework of mutational signatures.

Reference implementation: Indel segmentation

```
#include <Rcpp.h>
using namespace std;
using namespace Rcpp;

bool compareSegmentations(std::vector<int> v1, std::vector<int> v2){
  if(v1[3] > v2[3]){
    return true;
  }else if(v1[3] < v2[3]){
    return false;
  } else{
    if(v1[1] > v2[1]){
      return true;
    }else if(v1[1] < v2[1]){
      return false;
    }else{
      if(v1[2] < v2[2]){
        return true;
      }else if(v1[2] > v2[2]){
        return false;
      }else{
        if(v1[0] < v2[0]){
          return true;
        }else if(v1[0] > v2[0]){
          return false;
        }else{
          return false;
        }
      }
    }
  }
}

int ltrs(std::string::iterator str1_start,std::string::iterator str1_end,
         std::string::iterator str2_start,std::string::iterator str2_end){

  int increment_unit = std::distance(str1_start,str1_end);
  int coverage = 0;
  bool equal_unit=true; // assume all substrings are identical

  for(auto i = str2_start; i!=str2_end;){
    for(auto j = str1_start; j!=str1_end; j++,i++){
      equal_unit = equal_unit & (*i == *j);

      if(!equal_unit){
        break;
      }
    }

    if(!equal_unit){ // if is_equal_unit is false, break out.
      break;
    }
    coverage += increment_unit;
  }
  return coverage;
}

std::vector<int> segmentSingle(std::string &string,std::string &context){
  int string_size= string.size();
  std::vector<std::vector<int> > scores(string_size,std::vector<int>(4));
```

```
  int i = 0;
  for(auto unit_iter = string.begin()+1; unit_iter<=string.end();++unit_iter,++i){
    scores[i][0]=i+1;
    scores[i][1]=ltrs(string.begin(),unit_iter,unit_iter,string.end());
    scores[i][2]=string_size - scores[i][0] - scores[i][1];
    scores[i][3]=ltrs(string.begin(),unit_iter,context.begin(),context.end());
  }
  // in place sort
  std::sort(scores.begin(),scores.end(),compareSegmentations);
  return scores[0];
}

// [[Rcpp::export]]
Rcpp::DataFrame segment(std::vector<std::string> string, std::vector<std::string> context){
  // init vector
  int n_strings = string.size();
  std::vector<std::vector<int> > results(string.size(),std::vector<int>(4));
  // Apply segment_single via transform
  std::transform(string.begin(),string.end(),context.begin(),results.begin(),segmentSingle);
  // Init storage vectors
  std::vector<std::vector<std::string> > string_results(4,std::vector<std::string>(n_strings));
  std::vector<std::vector<int> > numeric_results(4,std::vector<int>(n_strings));
  // Transform results
  for(int i=0; i < n_strings; i++){
    // Transpose numeric results
    numeric_results[0][i]=results[i][0];
    numeric_results[1][i]=results[i][1];
    numeric_results[2][i]=results[i][2];
    numeric_results[3][i]=results[i][3];
    // Transform string results
    string_results[0][i]=string[i].substr(0,numeric_results[0][i]);
    string_results[1][i]=string[i].substr(numeric_results[0][i],numeric_results[1][i]);

string_results[2][i]=string[i].substr(numeric_results[0][i]+numeric_results[1][i],numeric_results
[2][i]);
    string_results[3][i]=context[i].substr(0,numeric_results[3][i]);
    // Divide internal_reps bases / unit_length , prime3_reps bases / unit_length
    if(numeric_results[0][i]!=0){
      numeric_results[1][i]=numeric_results[1][i]/numeric_results[0][i]; // internal_reps //
undivided
      numeric_results[3][i]=numeric_results[3][i]/numeric_results[0][i]; // prime3_reps //
undivided
    }
  }

  // Generate data frame
  Rcpp::DataFrame df_results = Rcpp::DataFrame::create(
    _["unit"]=string_results[0],
    _["unit_length"]=numeric_results[0],
    _["internal_rep"]=string_results[1],
    _["internal_reps"]=numeric_results[1],
    _["spacer"]=string_results[2],
    _["spacer_length"]=numeric_results[2],
    _["prime3_rep"]=string_results[3],
    _["prime3_reps"]=numeric_results[3],
    _["stringsAsFactors"] = false);

  return df_results;
}
```

```
segment_indels <- function(df){

  df <- segment(string = df$change,context = df$slice3)

  ## calculate original reps
  df$original_reps=ifelse(df$indel.type=='I',df$prime3_reps, ## if insertion --> TR
                      ifelse(df$indel.type=='D', ## if deletion:
                             ifelse(df$spacer_length==0, ## if S=0
                                    df$prime3_reps+df$internal_reps+1, ## TR + IR + 1
                                    df$prime3_reps ## else if S!=0 --> TR
                             ),
                             df$prime3_reps  ## if not ins / del --> TR
                      ))

  ## calculate microhomology length
  df$mh_length <- df$indel.length-df$spacer_length
  df$prime3_rep_length <- nchar(df$prime3_rep)
  df[df$mh_length>s$prime3_rep_length,"mh_length"] <-
                     df[df$mh_length>$prime3_rep_length,"prime3_rep_length"]

  return(df)
}
```

Supplementary References

1. Tanay, A. & Siggia, E.D. Sequence context affects the rate of short insertions and deletions in flies and primates. *Genome Biol* **9**, R37 (2008).
2. Alexandrov, L.B. *et al.* The repertoire of mutational signatures in human cancer. *Nature* **578**, 94-101 (2020).
3. Ye, K. *et al.* Systematic discovery of complex insertions and deletions in human cancers. *Nat Med* **22**, 97-104 (2016).
4. Carvajal-Garcia, J. *et al.* Mechanistic basis for microhomology identification and genome scarring by polymerase theta. *Proc Natl Acad Sci U S A* **117**, 8476-8485 (2020).
5. Kucab, J.E. *et al.* A Compendium of Mutational Signatures of Environmental Agents. *Cell* **177**, 821-836 e16 (2019).
6. Zou, X. *et al.* A systematic CRISPR screen defines mutational mechanisms underpinning signatures caused by replication errors and endogenous DNA damage. *Nat Cancer* **2**, 643-657 (2021).
7. Consortium, I.T.P.-C.A.o.W.G. Pan-cancer analysis of whole genomes. *Nature* **578**, 82-93 (2020).
8. Priestley, P. *et al.* Pan-cancer whole-genome analyses of metastatic solid tumours. *Nature* **575**, 210-216 (2019).
9. Turnbull, C. Introducing whole-genome sequencing into routine cancer care: the Genomics England 100 000 Genomes Project. *Ann Oncol* **29**, 784-787 (2018).

Supplementary Table Legends

Supplementary Table 1: CRISPR-edited RPE1 isogenic cellular models and their genotypes.

Supplementary Table 2: CRISPR gene-editing guide RNAs, HDR donor templates, and genotyping sequencing primers.

Supplementary Table 3: WGS coverage, *de novo* mutation count, and snv-to-indel ratio of experimental subclones.

Supplementary Table 4: Experimental gene-edit indel mutation catalogues and signatures in COSMIC-83 and 89-channel formats.

Supplementary Table 5: Experimental gene-edit single base substitution catalogues and signatures.

Supplementary Table 6: Replicative strand asymmetry analysis result.

Supplementary Table 7: Full 476 indel subtypes of re-defined indel taxonomy.

Supplementary Table 8: Indel examples of re-defined indel classification of small insertions and deletions (<100bp) for mutational signature analysis.

Supplementary Table 9: Tissue-specific indel signatures (InDs) of Genomics England seven cancer types (*n*=4,775).

Supplementary Table 10: 37 consensus indel signatures (InDs) in numerical format.

Supplementary Table 11: Conversion table from tissue-specific InDs to consensus InDs.

Supplementary Table 12: PRRDetect of GEL samples (*n*=4775).

Supplementary Table 13: Performance metrics of attempted prediction models using different input parameters.

Supplementary Table 14: PRRDetect feature coefficients.

Supplementary Table 15: Performance metrics of different algorithms for predicting PRR dysfunction in the validation cohort (*n*=1,351).

Supplementary Table 16: PRRDetect of ICGC breast samples (*n*=504).

Supplementary Table 17: PRRDetect of ICGC and Hartwig selected samples (*n*=1,335).
