# Supplementary material for: A redefined InDel taxonomy provides insights into mutational signatures
Source: Nat Genet. 2025 Apr 10;57(5):1132–41. doi: 10.1038/s41588-025-02152-y (PMC12081297; doi:10.1038/s41588-025-02152-y)
Supplement: Supplementary file 2 — Reporting Summary [file 41588_2025_2152_MOESM2_ESM.pdf]

nature portfolio

Corresponding author(s): Serena Nik-Zainal

Last updated by author(s): Feb 5, 2025

Reporting Summary

Nature Portfolio wishes to improve the reproducibility of the work that we publish. This form provides structure for consistency and transparency in reporting. For further information on Nature Portfolio policies, see our Editorial Policies and the Editorial Policy Checklist.

Statistics

For all statistical analyses, confirm that the following items are present in the figure legend, table legend, main text, or Methods section.

n/a	Confirmed	
☐	☒	The exact sample size (*n*) for each experimental group/condition, given as a discrete number and unit of measurement
☐	☒	A statement on whether measurements were taken from distinct samples or whether the same sample was measured repeatedly
☐	☒	The statistical test(s) used AND whether they are one- or two-sided<br>*Only common tests should be described solely by name; describe more complex techniques in the Methods section.*
☒	☐	A description of all covariates tested
☒	☐	A description of any assumptions or corrections, such as tests of normality and adjustment for multiple comparisons
☐	☒	A full description of the statistical parameters including central tendency (e.g. means) or other basic estimates (e.g. regression coefficient) AND variation (e.g. standard deviation) or associated estimates of uncertainty (e.g. confidence intervals)
☐	☒	For null hypothesis testing, the test statistic (e.g. *F*, *t*, *r*) with confidence intervals, effect sizes, degrees of freedom and *P* value noted<br>*Give P values as exact values whenever suitable.*
☒	☐	For Bayesian analysis, information on the choice of priors and Markov chain Monte Carlo settings
☒	☐	For hierarchical and complex designs, identification of the appropriate level for tests and full reporting of outcomes
☒	☐	Estimates of effect sizes (e.g. Cohen's *d*, Pearson's *r*), indicating how they were calculated

Our web collection on statistics for biologists contains articles on many of the points above.

Software and code

Policy information about availability of computer code

Data collection	We performed whole-genome sequencing of all experimental RPE1 samples generated in this study on Illumina Novaseq 6000 platform, generating 150 base pair paired-end reads, aiming for an average genome-wide sequence coverage of 25x.
Data analysis	De novo signature extraction and decomposition of mutational signatures was performed using SigProfilerExtractor (v.1.1.18), along with SigProfilerMatrixGenerator (v.1.2.4) available at https://github.com/AlexandrovLab/SigProfilerExtractor. Signatures were also extracted using signature.tools.lib (https://github.com/Nik-Zainal-Group/signature.tools.lib, v.2.4.4) and MuSiCal (https://github.com/parklab/MuSiCal, v.1.0.0) using default parameters. Experimental WGS short read data were were aligned to the human reference genome GRCh38 assembly using "bwa mem 0.7.17-r1188". Quality control and bioinformatic analysis of the WGS data was performed using "CaVEMan v1.13.15" for substitutions, "Pindel v3.2.0" for insertions/deletions. Structural rearrangement counts were low, copy number data were not informative, and hence not analysed. Experimental signature derivation was performed as described in doi: 10.1038/s43018-021-00200-0 and codes can be obtained from https://github.com/xqzou/COMSIG_KO and https://github.com/Nik-Zainal-Group/signature.tools.lib. The source code for clinical classifier, PRRDetect, can be obtained from https://github.com/Nik-Zainal-Group/PRRDetect. Indel segmentation and signature classification script can be accessed via https://github.com/Nik-Zainal-Group/indelsig.tools.lib.<br><br>For replicative strand analysis, intersectBed in BEDtools (v.2.26.0-114-g4c407ce) was utilized to identify mutations overlapping specific genomic features.

For manuscripts utilizing custom algorithms or software that are central to the research but not yet described in published literature, software must be made available to editors and reviewers. We strongly encourage code deposition in a community repository (e.g. GitHub). See the Nature Portfolio guidelines for submitting code & software for further information.

Data

Policy information about availability of data

All manuscripts must include a data availability statement. This statement should provide the following information, where applicable:

- Accession codes, unique identifiers, or web links for publicly available datasets
- A description of any restrictions on data availability
- For clinical datasets or third party data, please ensure that the statement adheres to our policy

Genomics England (GEL) cohort data (version 8) can be accessed via https://www.genomicsengland.co.uk/. Indels were called with Strelka v.2.4.7 using somatic calling mode. ICGC and TCGA WGS data were as published in https://doi.org/10.1038/s41586-020-1969-6 and can be obtained from https://dcc.icgc.org/releases/PCAWG. Hartwig metastasis WGS data can be obtained from Hartwig Medical Foundation through standardized procedures and request forms that can be found at https://www.hartwigmedicalfoundation.nl/en/appyling-for-data/.

Raw sequence files from the hTERT-RPE1 mutation accumulation experiment are deposited at the European Genome-Phenome Archive with accession numbers EGAD50000000209. Mutation call, variant data have been deposited at Mendeley: doi: 10.17632/3k2tpx9ssr.2. The curated data are available for general browsing from our reference mutational signatures website, Signal (https://signal.mutationalsignatures.com). Mutagen exposure data in human induced pluripotent stem cells were published and can be accessed via https://data.mendeley.com/datasets/m7r4msjb4c/2. Human iPS knockout data were obtained directly from doi: 10.1038/s43018-021-00200-0.

Primary data from the 100,000 Genomes Project, which are held in a secure research environment, are available to registered users. See https://www.genomicsengland.co.uk/research for further information or contact research-network@genomicsengland.co.uk. The results of RPE1 experimental signatures can be browsed at https://signal.mutationalsignatures.com/explore/main/experimental/experiments?study=7. InD signatures of the seven cancer types are accessible at https://signal.mutationalsignatures.com/explore/main/cancer/signatures?mutationType=3&study=7. All downstream analyses data and results are provided in the Supplementary Tables accompanying the study.

Research involving human participants, their data, or biological material

Policy information about studies with human participants or human data. See also policy information about sex, gender (identity/presentation), and sexual orientation and race, ethnicity and racism.

Reporting on sex and gender	n/a
Reporting on race, ethnicity, or other socially relevant groupings	n/a
Population characteristics	n/a
Recruitment	n/a
Ethics oversight	n/a

Note that full information on the approval of the study protocol must also be provided in the manuscript.

Field-specific reporting

Please select the one below that is the best fit for your research. If you are not sure, read the appropriate sections before making your selection.

☒ Life sciences ☐ Behavioural & social sciences ☐ Ecological, evolutionary & environmental sciences

For a reference copy of the document with all sections, see nature.com/documents/nr-reporting-summary-flat.pdf

Life sciences study design

All studies must disclose on these points even when the disclosure is negative.

Sample size	From a statistical standpoint, this was an exploratory study, and there were no pre-defined hypothesis tests for which sample-size power calculations would have been appropriate. The sample size was determined by the numbers of genes of interest, or by the number of tumor genomes in relevant cancer types represented by publicly available somatic mutation data, e.g., Sample size of GEL cohort was chosen based on the availability of whole genome sequencing of tumour/normal pairs in the Genomics England research environment.
Data exclusions	rom a statistical perspective, this was an exploratory study, and there were no pre-defined hypothesis tests for which pre-defined data exclusion criteria would have been appropriate. Therefore, no data were excluded from by our algorithms. Having said that, we did exclude four control samples for quantitative analyses. This was specified in Supplemental Table 1. The reasons for exclusion were mainly due to the culture doubling time differences. They were included in qualitative mutational signature spectra analysis for more stability, but not considered for mutation count/burden analyses.

Replication	Each experimental gene edit has at least 2-5 sub-clones as biological replicates per genotype. They were also all validated with in vivo cancer analyses.
Randomization	The question of allocation to experimental groups is not applicable to this study. No randomization was performed. All experimental samples were contrasted against an isogenic unedited/WT control.
Blinding	We applied the analysis algorithms to each and every gene edit in the dataset in exactly the same way and without any prior expectations about the desired outcome of the analysis. Therefore, blinding was not required.

Reporting for specific materials, systems and methods

We require information from authors about some types of materials, experimental systems and methods used in many studies. Here, indicate whether each material, system or method listed is relevant to your study. If you are not sure if a list item applies to your research, read the appropriate section before selecting a response.

Materials & experimental systems

n/a	Involved in the study	
☒	☐	Antibodies
☐	☒	Eukaryotic cell lines
☒	☐	Palaeontology and archaeology
☒	☐	Animals and other organisms
☒	☐	Clinical data
☒	☐	Dual use research of concern
☒	☐	Plants

Methods

n/a	Involved in the study	
☒	☐	ChIP-seq
☒	☐	Flow cytometry
☒	☐	MRI-based neuroimaging

Eukaryotic cell lines

Policy information about cell lines and Sex and Gender in Research

Cell line source(s)	The original hTERT RPE-1 are hTERT-immortalized retinal pigment epithelial cells derived by transfecting the RPE-340 cell line with the pGRN145 hTERT-expressing plasmid, which is commercially available from ATCC (https://www.atcc.org/products/crl-4000). This is a near-diploid human cell line of female origin with a modal chromosome number of 46 that occurred in 90% of the cells counted. The specific clone used in this study was originally generated from doi: 10.1038/s41586-018-0291-z. They were gifts from M. Tarsounas, (Department of Oncology, University of Oxford, UK).
Authentication	The cell lines were not authenticated in this study. However, we did have the whole genome-sequencing data and had matched SNP genotype profiles to confirm the cell line identities and their isogenicity.
Mycoplasma contamination	Stock cell line was tested negative for mycoplasma contamination when banked and used for the first time, but not tested again throughout the mutation accumulation experiment and subsequent single-cell subcloning steps.
Commonly misidentified lines (See ICLAC register)	Not applicable as none were used.

Plants

Seed stocks	Not applicable as none were used.
Novel plant genotypes	Not applicable as none were used.
Authentication	Not applicable as none were used.
